# Toward the Decipherment of Molecular Interactions in the Diabetic Brain

**DOI:** 10.3390/biomedicines10010115

**Published:** 2022-01-06

**Authors:** Maria Chomova

**Affiliations:** Institute of Medical Chemistry, Biochemistry and Clinical Biochemistry, Faculty of Medicine, Comenius University, Sasinkova 2, 81108 Bratislava, Slovakia; maria.chomova@fmed.uniba.sk

**Keywords:** diabetes, brain, insulin, ROS, mitochondria, proteostasis

## Abstract

Diabetes mellitus (DM) has been associated with cognitive complications in the brain resulting from acute and chronic metabolic disturbances happening peripherally and centrally. Numerous studies have reported on the morphological, electrophysiological, biochemical, and cognitive changes in the brains of diabetic individuals. The detailed pathophysiological mechanisms implicated in the development of the diabetic cognitive phenotype remain unclear due to intricate molecular changes evolving over time and space. This review provides an insight into recent advances in understanding molecular events in the diabetic brain, focusing on cerebral glucose and insulin uptake, insulin action in the brain, and the role of the brain in the regulation of glucose homeostasis. Fully competent mitochondria are essential for energy metabolism and proper brain function; hence, the potential contribution of mitochondria to the DM-induced impairment of the brain is also discussed.

## 1. Introduction

**Figure 1:**
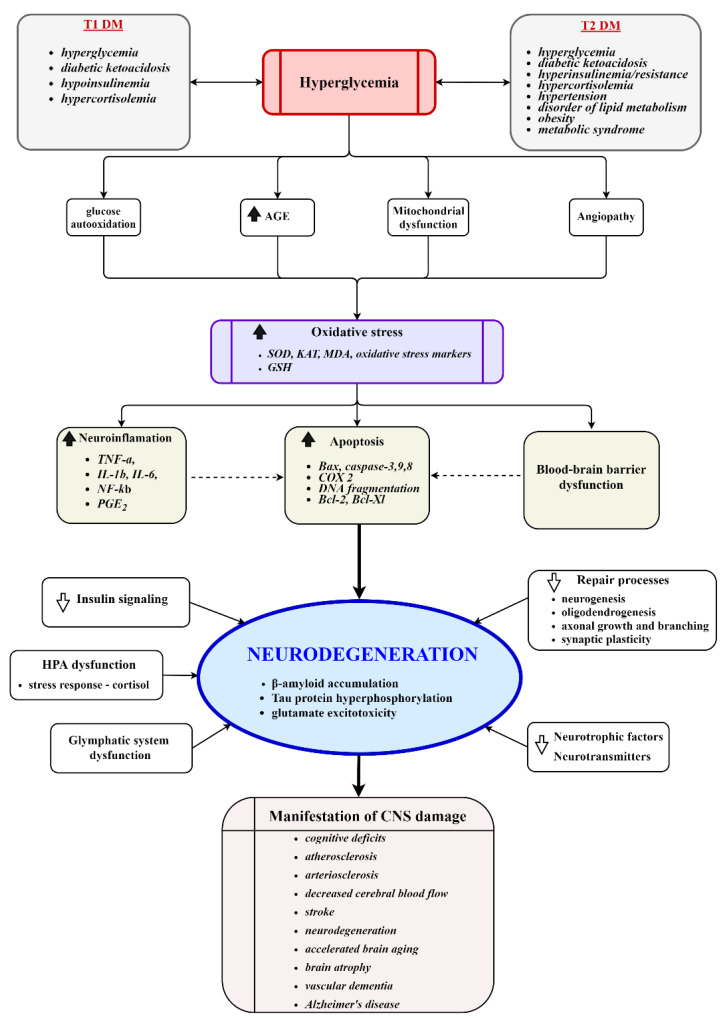
Key molecular events involved in the development of diabetic encephalopathy. Chronic hyperglycemia induces vascular injury, mitochondrial dysfunction, oxidative stress, neuroinflammation, dysfunction of the HPA axis, impairment of repair processes, reduced disposal of metabolic waste products, and activates apoptotic processes. The processes culminate in damage to the cerebral structure, neurodegeneration, and the manifestation of clinical symptoms. AGE, advanced glycation end products; SOD, superoxide dismutase; KAT, catalase; GSH, glutathione; HPA, hypothalamic–pituitary–adrenal axis.

Diabetic encephalopathies are accepted complications of DM [1]. Area-specific structural changes in the brains of diabetic patients, e.g., significantly reduced volumes of the hippocampus and prefrontal brain regions, higher rates of global cerebral atrophy, or the loss of white matter volume in the temporal lobe and inferior frontal triangle region, are the most significant changes reported by observational studies [2,3]. These changes co-occur with moderate alterations in the neurochemical profiles of N-acetyl aspartate, glutamate, myo-inositol, and choline in the white or grey matter of DM individuals and may be associated with impaired cognitive functioning [4]. The extent of metabolic (Figure 1) and cognitive alterations is determined by the interaction between disease and sensitivity of the brain to either a developmental phase (type 1—T1DM) or age (type 2—T2DM). The most evident decrement in T1DM patients is in the areas of general intelligence, psychomotor speed, mental flexibility, memory, and poor school performance. A cognitive change across the lifespan is greatest in those with an early onset of diabetes (under 6 years old) but the rate of further cognitive decline is slow—at least during the first 10 to 15 years after diagnosis [5]. Chronic hyperglycemia, microvascular complications, or recurrent episodes of hypoglycemia increase the risk of poorer cognition in older (>50 old) adults [6]. In T2DM, older adults most often show evidence of slowed information processing and poorer executive functions, and typically verbal and visual memory dysfunction, an impairment rarely associated with T1DM [7]. In elderly patients (>65 old), T2DM is associated with more severe forms of cognitive impairment. A recently published meta-analysis of 14 studies comprising over 2.3 million individuals and 102,000 cases of dementia concluded that individuals with T2DM are at a 60% greater risk of developing dementia compared with those without DM [8]. Whereas T2DM multiplies the risk of vascular dementia, the increased risk of Alzheimer’s disease is still controversial [9,10]. 

Likewise, the results of multivariate analyses scoring the impact of prediabetes and metabolic syndrome on cognitive performance are controversial, but metabolic syndrome or impaired fasting glucose may be a risk factor for cognitive dysfunction [11]. Despite intensive research, detailed knowledge of factors and cellular mechanisms contributing to the development of the diabetic cognitive phenotype remains extremely difficult because of the multifactorial and chronic character of the disease and the biomedical and psychosocial heterogeneity of diabetic individuals.

In this work, recent advances in understanding the pathophysiological mechanisms of DM-linked impairment of the brain are reviewed. The work does not attempt to be fully comprehensive; it is aimed at the most important and interesting aspects of cellular processes that can progress to brain dysfunction and cognitive decline. Glucose is an essential energy source for the brain; thus the first section deals with cerebral glucose uptake and the participation of the brain in the regulation of glucose homeostasis. In the next section, attention is given to insulin and its role in the brain. Proper mitochondrial function is an inevitable prerequisite for the physiological functioning of the neurons, hence a potential contribution of mitochondria to CNS damage under diabetes is also discussed.

## 2. Cerebral Glucose Uptake in Diabetes

In healthy individuals, the brain glucose level rises in a linear fashion with a rising plasma glucose level [12]. Glucose uptake into the brain depends on two key factors: on the concentration gradient between glucose in the blood–brain interstitium and the expression of GLUT1, the key glucose transporter for transport across the blood–brain barrier (BBB). Its endothelial protein concentration is regulated by circulating glucose concentrations, and it is under both transcriptional and post-transcriptional control [13]. The largest proportion of glucose enters astrocytes due to the release of a neurotransmitter, namely, glutamate. According to the astrocyte–neuron–lactate shuttle model, astrocytes respond to glutamatergic activation and Na^+^-dependent uptake of glutamate from the synaptic cleft into astrocytes by increasing the rate of glucose uptake to meet the energy demands of activated Na^+^/K^+^ ATPase. The increased glycolytic flux results in the production and release of lactate from astrocytes, which is then available as a fuel for neurons [14]. Neurons can also absorb glucose via the GLUT3 transporter and metabolize it in the process of glycolysis and oxidative phosphorylation [15]. GLUT3 is often co-expressed with the insulin-sensitive glucose transporter GLUT4 that is involved in local ATP production for firing neurons [16]. Na^+^–glucose cotransporters SGLT are also of physiological importance to cerebral glucose transport, e.g., SGLT1 in the BBB may be involved in the adjustment of glucose concentration in the brain interstitium. High expression of SGLT1 was also observed in brain areas involved in learning, memory formation, energy expenditure, feeding behavior, and regulation of glucose homeostasis [17]. Furthermore, additional transporters may also participate in glucose uptake into the brain.

In DM, blood glucose fluctuations are a typical feature that might affect glucose uptake into the brain by modifying transporter expression, transport kinetics, or BBB permeability. Adequate conclusions are currently lacking, as the studies struggle with controversial results, probably due to the large heterogeneous cerebral microvascular bed. Generally, the glucose transport to the brain is regulated in the endothelial capillary wall of the BBB in response to neuronal activity and involves regulation of blood supply, adjustment of driving forces for transport, and regulation of the expression of glucose transporters. Some evidence suggests a key role for the transcription factor HIF-1α in normal glucose metabolism [18]. Earlier animal studies have suggested that chronic hyperglycemia decreased glucose transport into the brain due to the downregulation of GLUT1 at the BBB, while others indicated no changes. Some investigators have observed an apparent discordance between the abundance of GLUT1 mRNA and protein in the BBB, suggesting a DM-induced defect in the transporter translation [19]. It is well known that obesity is a risk factor for insulin resistance and DM. High-fat (HF) diet experiments showed that short-term HF feeding led to the suppression of brain glucose uptake in mice due to the saturated fatty acid-induced impairment of GLUT1 activity and GLUT1 downregulation in the BBB. Surprisingly, prolonged feeding restored the transporter expression parallel with an increase in expression of vascular endothelial growth factor (VEGF) in macrophages at the BBB [20]. Increased VEGF production may represent a compensatory effect aimed at restoring cerebral glucose metabolism and preventing cognitive dysfunction and neurodegeneration. This adaptive response probably involves multiple mechanisms, such as inflammatory signals from perivascular macrophages or a reduction in parasympathetic tone, which is well known to contribute to the activation of inflammation in obesity [21].

Reduced brain glucose uptake associated with impaired glucose tolerance and insulin resistance has also been observed in mice with brain-specific knockout of the insulin-dependent GLUT4 transporter, suggesting a role for the transporter in the homeostatic regulation of glucose [22].

In humans, studies focusing on T1DM have observed similar glucose uptake and cerebral concentrations under normo-, hyper-, and hypoglycemic conditions, indicating that the glucose-dependent regulation of GLUTs seems to be intact [23]. Measurement of glucose levels in the brain of obese individuals and T2DM by magnetic resonance spectroscopy noticed an impaired cerebral energy gain upon a glucose load in these individuals [24]. Lower brain glucose increments were also noticed in the occipital lobe in participants with obesity and poorly controlled T2DM in response to acute hyperglycemia invoked by hyperglycemic clamp [25]. In view of these findings, a blunted rise in brain glucose levels in T1DM, T2DM, and obese subjects might represent an adaptive response of the brain aimed at decreasing glucose entry to prevent hyperglycemia-induced adverse effects.

Notably, there may be regional differences in cerebral glucose uptake due to heterogeneous brain architecture and area-specific functions. Thus, further studies focusing on brain regions involved in energy homeostasis, food intake, or cognitive functions are needed to gain more insight into brain glucose uptake under diabetes and other metabolic diseases.

## 3. Brain Role in Maintenance of Glucose Homeostasis

The islet-centered view of the control of glucose homeostasis via the insulin action on insulin-sensitive tissues and the key role of the liver to govern blood glucose represent the canonical explanation of regulatory mechanisms of glucose homeostasis in the body. However, recent research brings growing evidence of more complex regulation of systemic glucose. This regulation employs a highly coordinated interplay between the brain glucoregulatory system and pancreatic islets to set normal glucose levels. This circuit system can modulate blood glucose levels by both insulin-dependent and insulin-independent mechanisms [26,27].

### 3.1. Insulin-Dependent Mechanism

**Figure 2:**
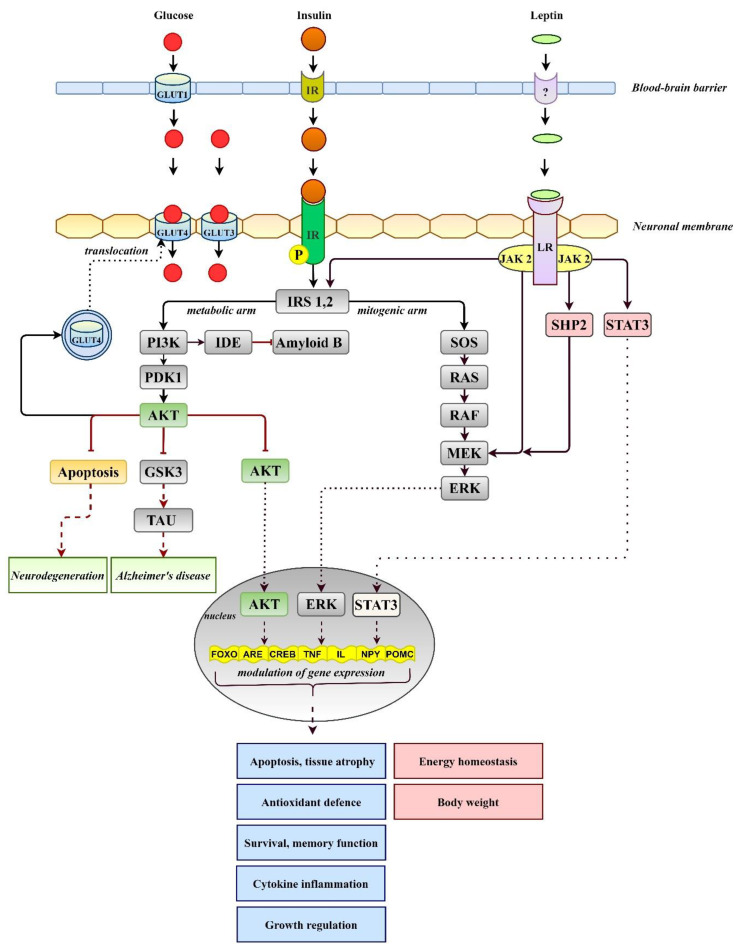
Simplified overview of insulin and leptin signaling. Insulin triggers activation of AKT in the metabolic arm, which modulates antioxidant defense, memory function, and growth- or cytokine-induced inflammation. Leptin after binding to its receptor (LR, long isoform) activates gene expression via phosphoinositol 3 kinase (PI3K), an activator of transcription (STAT3), and extracellular signal-regulated kinase (MAPK/ERK) pathways. IR, insulin receptor; IDE, insulin-degrading enzyme; GLUTs, glucose transporters; GSK3, glycogen synthase kinase-3; PDK1, 3-phosphoinositide-dependent protein kinase; SHP2, sulfhydryl-domain containing protein tyrosine phosphatase; TAU, tau protein.

In the direct mechanism (Figure 2), the brain can govern adaptive responses to modulate systemic glucose. As evidenced by animal experimental approaches, administration of glucose either through the 3rd ventricle, the arcuate nucleus, or the brain vasculature without changing systemic glycemia led to the activation of hypothalamo-hepatic and hypothalamo–pancreatic axes and physiological responses, such as glycogenesis and insulin secretion [28,29]. Alike, insulin infusion into the 3rd cerebral ventricle of rats suppressed hepatic glucose production independent of circulating levels of insulin and other glucoregulatory hormones [30]. However, insulin infusion into hypothalamic areas with the knockdown of insulin receptors failed to suppress the glucose production in the liver [31]. 

Non-hypothalamic brain regions also seem to be involved in the extrahepatic control of glucose production because insulin-activated Erk1/2 signaling in the dorsal vagal complex of the hindbrain resulted in the suppression of hepatic glucose production [32]. Brain participation in hepatic glucose production was also assumed in the TLKO mice, the model of the liver-specific deletion of key signal transduction molecules AKT and FOXO1. In this model, hepatocytes are unable to respond to the direct effect of insulin, but systemic administration of insulin constrained hepatic glucose production, thus pointing to an extra-hepatic effect of insulin [33]. Another piece of evidence for a neuronal circuit regulating blood glucose was added following experimental findings of unchanged glucose tolerance, decreased insulin secretion, but increased insulin sensitivity in cold-exposed rats [34].

These findings support the hypothesis that pancreatic islets operate with a high degree of autonomy under physiological conditions, so a brain intervention required to regulate glucose homeostasis is minimal. As pathological inputs deviate the glucose level out of normal range, the role of the brain becomes more evident, and activation of the brain’s homeostatic function ensures glucose availability as the energy source.

### 3.2. Insulin-Independent Mechanism

In addition to direct insulin-dependent regulation, the brain appears to have the ability to regulate blood glucose levels independently of insulin. The idea of insulin-independent regulation of blood glucose came from observations of the antihyperglycemic action of the adipocyte hormone leptin. The hormone is able to inhibit food intake and increase energy expenditure and glucose uptake in muscle and brown adipose tissue to suppress the production of glucagon and corticosterone or inhibit hepatic glucose output (for more detail, see [35]). The pleiotropic effects of leptin on various tissues suggest that modulation of a single pathway is insufficient to restore glucose homeostasis. Once bound to receptors, the leptin effects may be inhibitory or stimulatory and may be partly mediated by direct leptin signaling (Figure 2) within peripheral tissues. However, the ability of leptin to lower blood glucose appears to be mediated primarily by neuronal pathways within the CNS, particularly in the hypothalamus, as well CNS-mediated effects on the periphery. Amelioration of glycemic control during four months of leptin therapy in patients with DM and severe lipodystrophy has been reported [36]. In T1DM models, adenoviral-induced hyperleptinemia corrected severe hyperglycemia, despite the absence of insulin, via suppressing glucagon action in the liver and enhancing the insulinomimetic action of the insulin-like growth factor in muscle [37]. The continuous infusion of leptin at supraphysiological doses directly into the brains of T1DM rats led to blood glucose normalization [38]. When leptin administration was stopped, hyperglycemia was quickly reestablished in the absence of changes in insulin, indicating the central effect of leptin on the modulation of glucose metabolism [39]. Of note, severe insulin deficiency is associated with severe leptin deficiency, whereas insulin treatment reverses hypoleptinemia [40]. Given the positive insulin–leptin relationship, high levels of leptin are required to compensate for severe insulin deficiency. Thus, central leptin infusion clearly points to the brain’s participation in the maintenance of glucose homeostasis by activating brain leptin receptors and cellular signal pathways. The detailed cellular mechanisms of the central antidiabetic effect of leptin are still elusive. It was proposed that binding the hormone to its receptor activates three main signaling pathways (Figure 2): (a) insulin receptor substrate 2/phoshatidylinositol 3-kinase (IRS2/PI3K), (b) SH2-containing protein tyrosine phosphatase 2/MAPK (SHP2/MAPK), and (c) signal transducer and activator of transcription (STAT3) pathways [41]. Stimulation of the SHP2/MAPK pathway may be critical in modulating glucose metabolism because SHP2 deficiency in proopiomelanocortin (POMC) neurons abolished leptin’s ability to reduce blood glucose levels [42]. Moreover, an intact brain melanocortin system, particularly POMC neurons and melanocortin 4 receptors, is inevitable with the chronic antidiabetic effects of leptin as well as counterregulatory responses to hypoglycemia [43]. The role of the autonomic nervous system was also suggested since skeletal muscle denervation and adrenergic receptor blockade significantly attenuated leptin-induced increases in peripheral glucose uptake [44]. The involvement of the hypothalamic–pituitary–adrenal (HPA) axis was also investigated but hypophysectomy did not prevent or attenuate leptin’s anorexic and antidiabetic effects in streptozotocin-diabetic rats [45].

An increased interest in the brain participation in glucose homeostasis regulation was boosted by observations of the unexpected glucose-lowering effects of the fibroblast growth factors FGF1, FGF19, and FGF21 in obese and diabetic animals. The ability of FGFs as hormones with autocrine, paracrine, or endocrine effects to trigger signaling in the brain depends on their local expression, the presence of their receptors, or their ability to cross the blood–brain barrier. While FGF1 and the FGF receptors are differentially expressed in several parts of the brain, no expression of FGF19 and FGF21 has been found in the brain so far [46]. FGF19 is kept mainly in the circulation, but FGF21 can easily enter the brain [47]. It was shown in T1DM rat models that intracerebroventricularly administered FGF1 and FGF19 suppressed the HPA axis, resulting in a 60% reduction in hepatic glucose production, hepatic acetyl CoA content, whole-body lipolysis, and a decrease in plasma ACTH and corticosterone concentration. These effects were abrogated by an intra-arterial infusion of corticosterone [48]. Another study [49] also reported that a low dose of FGF1 administered intracerebroventricularly resulted in sustained diabetes remission in mouse and rat models of T2DM, but it did not reduce corticosterone levels, suggesting that the role of the HPA axis in diabetes remission is questionable. Since FGFs can be induced by multiple forms of mitochondrial dysfunction, they have been suggested to be mitokines [50]. Mitokines are hypothesized to be involved in the non-cell-autonomous signaling pathway aimed at activating the mitochondrial unfolded protein response, part of the integrated stress response directed to restore perturbed cell homeostasis. The role of FGFs in the central regulation of glucose homeostasis is surely interesting, but full significance will only become apparent in the future.

In summary, evidence suggests that glucose homeostasis is governed by a neuromodulatory circuit, in which the brain, islets, and peripheral tissues work in cooperation to adjust and regulate glycemia. In order for the system to work properly, intact brain sensing of the circulating glucose must be preserved [51]. A defect in the brain sensing of blood glucose can alter the circuit balance and set higher blood glucose levels (hyperglycemia) to ensure the energy requirements of the brain. It is likely that every part of the circuit disposes of potential, partially compensating for the failure of the other; however, the capacity overload of the system can result in its inability to maintain the physiological range of blood glucose, leading to diabetes.

## 4. Insulin and the Brain

As mentioned in the previous section, the brain insulin-dependent mechanism governs glucose homeostasis. To do this, insulin must enter the CNS and activate cellular signaling pathways. Despite a debate regarding local synthesis in the brain, brain insulin originates predominantly from the periphery, where its level fluctuates depending on physiological or pathological states. The restricted permeability of the BBB and the size of the molecule do not allow insulin to enter the CNS directly. It enters the brain mostly through receptor-mediated transcytosis after binding to insulin-binding sites of the BBB endothelial cells [52]. The insulin transport is not uniform between brain regions but varies depending on requirements and the number of insulin receptors (IRs) in these regions [53].

It is not clear whether the insulin transporter is a separate protein or a classic receptor. The receptor can exist in A and B isoforms [54]. Both isoforms have a similar binding affinity for insulin but insulin-like growth factor 2 (IGF-2) and proinsulin are bound only by IR-A. IR isoforms can also form hybrid heterodimers with the IGF-1 receptor (IGF-1R) and can have varying post-translational modifications leading to further diversity of insulin action [55]. Brain neurons appear to predominantly express the IR-A isoform, which mediates mostly non-metabolic effects, such as those implicated in motivating, rewarding, learning, and memory [56]. In contrast, astrocytes express the IR-B isoform, which participates in metabolic effects. Astrocytic IR ablation reduces glucose-induced activation of hypothalamic POMC neurons and impaired physiological responses to changes in glucose availability, demonstrating that astrocytic IRs are required for glucose and insulin entry through the BBB [57].

Different roles of the IR isoforms appear not to be exclusive determinants of insulin/IGF actions. Evidence suggests that both the IR-A/IR-B ratio and hybrid receptors may play an important role in final effects triggered by hormone binding. The IR expression is tightly regulated at three molecular levels by mechanisms not to be fully understood yet. IR regulation takes place at the promoter level in a developmental- and tissue-specific manner [58] at a posttranscriptional level by translation regulation of internal ribosome sites and alternative splicing of pre-mRNA and by microRNAs [59]. The relative protein abundance of the IR isoforms is set by differences in maturation processes that engage different convertases acting in the Golgi compartment [60]. Additional levels of complexity in IR regulation incorporate, for example, mechanisms of internalization and degradation of hormone-occupied receptors or binding of different proteins to IRs [61,62]. To illustrate, an increase in the IR-A isoform due to increased endocytosis and degradation of total IR protein or increase in hybrid receptors was observed at peripheral tissues in obesity, diabetes, cancer, or aging [63,64]. In the brain, the highest distribution of the IRs and IGF-1R was found in the olfactory bulb, hypothalamus, cortex, cerebellum, and hippocampus. Experimental and clinical studies have already reported on reduced levels of IRs, IGF-1 receptors, and central insulin resistance in DM and AD subjects, but data on the shift in the ratio of the IR-A/IR-B and hybrid receptors in the CNS are still missing [65]. Thus, alterations in the IR-A/IR-B ratio and hybrid receptors may be assumed to contribute to central insulin resistance, unbalanced insulin/IGF signaling, development of diabetic cognitive phenotype, and neurodegeneration.

Endothelial IRs are considered to be a key entry point for insulin into the brain, hence their role in insulin transport and signal cascade has been studied on several experimental levels. The initial studies in brain capillaries have brought evidence of the reduced IRs in obesity and hyperinsulinemia as well as increased endothelial binding and insulin transport through the BBB in experimental-induced DM [66,67]. Later studies in several genetic models with knockout receptors have found that the loss of IRs and IGF-1Rs on vascular endothelial cells (VENIRKO, VENIFARKO mice) did not disturb the structural integrity of the BBB [68]. In EndoIRKO mice, however, the loss of endothelial IRs led to delayed onset of receptor activation in the periphery, in the hypothalamus, hippocampus, and prefrontal cortex, suggesting impaired insulin delivery through the vascular barrier as well as central insulin resistance. Intact insulin signaling was sustained in the olfactory region, suggesting the hormone’s ability to circumvent the BBB and reach the brain [69]. It has been shown in T1DM-rats or AD mouse models that both acute and chronic intranasal insulin treatment reduces β-amyloid levels and repairs insulin signaling through the downregulation of tau kinases and alleviates cognitive deficits [70]. Currently, the intranasal approach is being used in short-term clinical trials to test improvements in brain energy levels, memory, and cognition deficits, but its long-term effect on energy and systemic metabolism is unclear [71,72].

Findings from endothelial cell cultures also indicate that IRs participate in insulin transcytosis, but the transport may be affected by specific factors and conditions, e.g., L-glutamate, inhibition of NO-synthase, high-fat diet, hypertriglyceridemia, or elevated plasma levels of the intestinal hormone cholecystokinin [73,74,75]. Some studies also point to insulin transport by the IR-independent transport and by alternative routes [76,77].

Besides acting as insulin transporters, IRs can also operate as classical signal transduction receptors and affect the BBB permeability by modulating the expression/activity of transmembrane proteins involved in the formation of brain endothelial junction complexes. Increased BBB permeability has been reported in experimental diabetic animal models [78]. Insulin also impacts various transporters in the BBB, as the receptor for advanced glycation end products, low-density lipoprotein receptor-related protein 1, ATP-binding cassette transporter family members, and those regulating the efflux of β-amyloid peptide out of the cerebral tissue, suggesting its implication in the development of Alzheimer’s pathology [79,80]. In AD patients, reduced insulin levels in the brain and cerebrospinal fluid (CSF), despite elevated plasma insulin levels, insinuate impaired transport of insulin across the BBB [81]. In those and DM patients, the decreased CSF insulin levels correlate with poorer cognitive performance [82]. Of note, the levels of insulin in the CSF and circulation are closely correlated with whole-body insulin sensitivity in insulin-sensitive humans but not in insulin-resistant humans [83]. The CSF/plasma insulin ratio is reduced in obesity, and age-dependent changes in the ratio are also observed in the elderly [84].

Fully functional astrocytic IRs are also required for appropriate insulin and glucose entry into the brain. Proper insulin signaling in astrocytes plays a key role in effective hypothalamic neuronal responses and regulation to appropriately counteract fluctuations in systemic glucose availability. Insulin action in astrocytes also regulates gliotransmission and modulates behavior [85].

Recent research has started to reveal the complex role of IRs in the CNS, though many issues still need to be addressed. Deciphering the role of the brain IRs in detail will provide the opportunity to modulate IR activity in a personalized and disease-specific context and will open a new avenue in the treatment of diabetic encephalopathology and other CNS pathologies.

## 5. Insulin–Mitochondria–ROS Interplay

Insulin action is inevitably linked to proper mitochondrial function, and, not surprisingly, aberrant mitochondria in the brain are connected to insulin resistance, metabolic syndrome, diabetic encephalopathies, neurodegenerative diseases, and aging. Alterations in electron transfer chain (ETC) function, energy metabolism [86], mitochondrial biogenesis, and fission [87], or an evident positive effect of insulin sensitizers on mitochondrial functions [88], are the most reported observations in many diabetic, obesity, or neurodegenerative studies and point to insulin–mitochondria interplay. One insulin–mitochondria interaction is “redox priming” as an intermediate phase in which oxidative modifications of sensitive cysteine residues facilitate insulin-induced receptor autophosphorylation. In neurons, insulin stimulation has been shown to generate a spike in mitochondrial H_2_O_2_ and this signal preceded activation of the IRs [89]. Although IR autophosphorylation seems to be insulin-dependent in nature, it did not occur until the H_2_O_2_ signal exceeded the threshold, even in the presence of high insulin concentrations. Moreover, the signal was ultra-sensitive to H_2_O_2_ scavenging, suggesting that a pathological increase in H_2_O_2_ scavenging antioxidant enzymes may also limit insulin signaling in the brain. Since IRs are concentrated at synapses that become enriched with mitochondria in the period of synaptic activity, a disturbance in the redox activation of IRs may represent another factor contributing to insulin resistance and cognitive impairment.

Any exogenous or endogenous stresses can perturb mitochondrial function, ROS production and ultimately impact brain energy metabolism. Neurons are especially vulnerable to mitochondrial stress since the energy stress-induced diversion of the physiological metabolic program of preferential utilization of glucose for regeneration of glutathione in the pentose phosphate cycle to the glycolytic pathway can weaken the antioxidant defense of neurons [90,91]. The hypothesis of excessive ROS production in DM is widely supported by evidence of damage to proteins, lipids, and DNA [92,93,94]. However, a significant decrease in mitochondrial membrane potential (Δψ_m_), mitochondrial respiration, the enzymatic activities of the respiratory chain, and energy levels were reported in the cortex and hippocampus of diabetic rodent models [86,93,95], diabetic sensory neurons [96], or diabetic islets from humans and rodent models [97,98]. On the contrary, increased ROS signaling and membrane hyperpolarization have been found in peripheral diabetic cell cultures [99]. Considering a widely accepted idea of a positive correlation between mitochondrial ROS production and ETC activity/Δψ_m_, published experimental data appear to be less conclusive. Besides numerous variations in experimental design, this inconsistency probably reflects many pitfalls of ROS measurement, especially in clearly assigning their origin to mitochondria [100].

Mitochondria are an integral part of the cellular network of adaptive and defensive responses aimed at sensing and adapting to alterations in nutrient supply or at limiting oxidative damage. In the brain, the expression and distribution of uncoupling proteins (UPCs) UCP2, UCP4, and UCP5 were induced by metabolic and oxidative challenges, suggesting the relevance of mitochondrial uncoupling to the control of neuronal, neuroendocrine, and autonomic responses [101]. Within these proteins, UCP2 is expressed abundantly in various brain areas and its key role in neuroprotection, the development of cognitive ability, resistance to anxiety, or hippocampal monoamine transmission, has been suggested [102,103]. The UCP2 uncoupler appears to be an important negative regulator of β-cell insulin secretion on the periphery, suggesting its role in the loss of glucose responsiveness in obesity-related T2DM [104]. Interestingly, the allele combination of IGF1R/IRS2/UCP2 was associated with a decreased all-cause mortality risk and with increased longevity, suggesting the combined effect of these genes on energy metabolism and the age-related metabolic remodeling capacity [105]. Observation of the downregulated UCP2 expression in rat T2DM hippocampus also indicates that its neuroprotective effect might be absent from the diabetic brain [106]. Genipin-induced inhibition of UCP2 activity not only downregulated its protein expression and enhanced ROS production in mice’s primary cortical neurons exposed to glucose fluctuation but also reduced mitochondrial biogenesis and led to the loss of neuronal synaptic integrity and cell viability. Concomitantly, inhibition of UCP2 function and the increase in metabolic and oxidative stress were compensated for with increased UCP4 expression, pointing to activated mitochondrial hormetic responses to uphold cell survival [107].

The insulin–mitochondria interplay is apparently observable in the processes of activating the mitochondrial stress response, as described in the next section.

## 6. Mitochondrial Proteostasis in the Diabetic Brain

**Figure 3:**
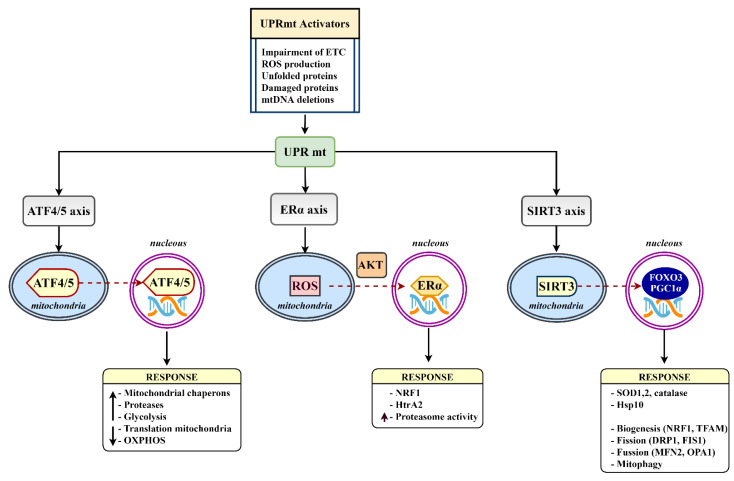
The mammalian UPRmt axes. Various UPRmt activators initiate gene expression via the ATF4/5, Erα, and SIRT3 branches. Triggered signaling pathways lead to a number of mitoprotective outcomes aimed at restoring mitochondrial homeostasis. ATF4/5, activating transcription factor 4/5; ERα, estrogen receptor alpha; DRP1, dynamin-related protein 1; FIS1, mitochondrial fission 1 protein; FOXO3, forkhead box protein O3; HTRA2, HtrA serine peptidase 2; MFN2, mitofusin 2; NRF1, nuclear respiratory factor 1; OPA1, mitochondrial dynamin-like GTPase; PGC1α, peroxisome proliferator-activated receptor-gamma coactivator-1 α; SIRT3, sirtuin 3; TFAM, mitochondrial transcription factor A.

Metabolic disturbances in DM, such as ETC dysfunction, ROS-mediated oxidative stress, or neuroinflammation invade cellular and mitochondrial proteomes. When the proteome alters its properties, damaged and misfolded proteins and unassembled precursors can accumulate and provoke proteotoxic stress. In response to mitochondrial proteotoxic stress, the cell activates an adaptive program referred to as the mitochondrial unfolded response (UPRmt). The program, as a component of the cellular stress response pathways, represents the signaling pathway where various stress stimuli, such as misfolded and accumulated proteins, mitochondrial DNA mutations, inhibition of mitochondrial chaperones and proteases, alterations in mitochondrial dynamics, or metabolic and oxidative stress, elicit a nuclear transcriptional response to reestablish cellular and mitochondrial homeostasis [108]. Of note, several mitochondrial stress responses can be induced by other factors without any apparent connection with mitochondrial protein misfolding. A certain degree of similarity to the UPRmt can be present in these responses due to overlapping pathways, suggesting the complex nature of stress response regulation [109]. Transcription of stress-induced target genes seems to be epigenetically modulated by histone 3-specific methylation and since DNA methylation and histone posttranslational modifications differ in specific brain regions, the UPRmt may also differ in distinct types of neuronal cells [110]. Proper activation of the UPRmt supports metabolic health and increases the lifespan; however, if sustained chronically it can lead to disease, including obesity, T2D, and neurodegenerative disorders. The coordinated action of the three UPRmt axes, the canonical ATF4/5, Erα, and SIRT3 axis (Figure 3), after activation leads to increased production of chaperons, protein folding, antioxidant capacity, and protein quality control [111].

The upregulation of mitochondrial chaperones and co-chaperones, mainly the group of heat shock proteins (e.g., Hsp60, Hsp70, Hsp10, HSC20, DNAJA3) and proteases (e.g., HtrA2, ClpP, Lonp1), constitutes the strongest cell response to stress. The Hsp response is predominantly cytoprotective because chaperones have the potential to attenuate pathology by the clearance of aggregated proteins, e.g., amyloid proteins. They also prevent further aggregation by inhibiting the nucleation and elongation processes of cross-seeding and facilitating cellular repair and defense mechanisms [112]. In DM, the Hsp-response is weakened and was shown to positively correlate with dysfunctional insulin signaling. In this mechanism, DM-associated increases in glucose synthase kinase-3 activity lead to abnormal phosphorylation of heat shock transcriptional factor 1 (Hsf1). Phosphorylated Hsf1 is less efficient at binding to the Hsp-transcription element and quenching the stress-induced transcriptional activity, decreasing the level of Hsp proteins [113]. The downregulation of the mitochondrial chaperone Hsp60 due to a lack of leptin signaling has been shown to be sufficient to induce hypothalamic insulin resistance in a T2DM murine model [114]. In the model of high fat diet-induced hypothalamic insulin resistance, a disrupted mitochondrial stress response led to mitochondrial dysfunction, excessive autophagy, and increased weight gain. Short-term intranasal insulin application restored expression of Atf4, Chop, Hsp60, Hsp10, ClpP, and Lonp1, suggesting that hypothalamic insulin/IGF1 signaling regulates mitochondrial stress response and ensures proper mitochondrial function [115]. Reduction in co-chaperone Hsp10, which modulates Hsp60 activity, was also sufficient to cause hypothalamic insulin resistance with acute liver insulin resistance, decreases in subunit protein levels of the ETC complexes, and mitochondrial dysfunction in T2DM mice. Interestingly, Hsp10 knockdown in murine hypothalamic cells increased saturated fatty acids (FA) and decreased monounsaturated FA content [116]. The shift from unsaturated FAs to saturated FAs in cardiolipin, the inner mitochondrial membrane phospholipid essential for the proper function of the respiratory enzymes and the assembly of the ETC into supercomplexes, was also observed in the brain cortex of streptozotocin-rats [93]. Elevated saturated FAs were also reported in patients with Hsp60 deficiency, metabolic syndrome, and in the cerebrospinal fluid of humans with obesity [117,118]. It is currently unclear how Hsp interferes with FA metabolism.

Several studies have also suggested a link between mitochondrial stress and neuroinflammation via Hsp60′s interaction with Toll-like receptors, which leads to the production of proinflammatory mediators, such as TNF-α, IL-1β, IL-6, and IL-8 [119,120]. Hyperglycemia-linked neuroinflammation in the CNS plays a key role in the development of vascular dementia in diabetic patients. Of note, Hsp60 holds many functions and occurs not only inside mitochondria but also in other intracellular locations, and it may be released from a cell too. Extracellular secretion of Hsp60 via exomes, which play an important role in cell-to-cell communication, has been documented in various inflammatory diseases including DM, suggesting that neuroinflammation could spread to neighboring neuronal cells, such as astrocytes [121,122].

Neuroinflammatory processes, a result of mitochondrial impairment, were also noticed in the model of metabolic syndrome (MS), a precondition for obesity, DM, and cardiovascular diseases. An important feature of MS is the deficiency of silent information regulator sirtuin 3 (Sirt3), the mitochondrial member of the group of NAD+-dependent lysine deacetylases. Deacetylases control a wide range of cellular processes, among them, Sirt3 controls energy metabolism processes, antioxidant defense, and mitochondrial dynamics [123]. The importance of sirtuins for cell homeostasis is highlighted by their engagement in the UPRmt stress response axis. In the brain of mice with MS, Sirt3 deficiency led to impaired mitochondrial respiration, downregulation of mitochondrial fission proteins Mfn1, Mfn2, elevated levels of brain IL-1β, and inflammasome formation [124]. Sirt3 deficiency-induced hyperacetylation of the mitochondrial proteome was also shown to spoil glucose metabolism, preferentially at the Krebs cycle, disarrange metabolic coupling between neurons and astrocytes, and decrease neurotransmitter synthesis [125]. In the mouse model of comorbid Alzheimer’s disease with amyloid pathology and MS, Sirt3 deficiency aggravated insulin resistance, glucose intolerance, amyloid plaque deposition, neuroinflammation, and microgliosis, suggesting that MS may interact with amyloid pathology during the early cellular phase of Alzheimer’s disease [126]. Thus, SIRT3 decline induced mitochondrial dysfunction and neuroinflammation in chronic metabolic diseases, such as DM, may be important participants in the cascade of molecular processes resulting in proteotoxic stress, neuronal cell damage, and late-life cognitive decline.

Healthy and fully functional mitochondria are maintained by unique equilibrium among the processes of mitochondrial biogenesis, removal of damaged mitochondria by mitophagy, and mitochondrial dynamics, which are regulated by mitofusins Mfn1, Mfn2, Drp1, and OPA1. Mitochondrial dynamics in the brain are associated with feeding, glucose homeostasis, and whole-body metabolism, and disorders of mitochondrial fission–fusion proteins are observed in obesity, DM, and neuroinflammation [127]. Mfn1 has recently emerged as a nutrient sensor in POMC neurons that influences whole-body glucose metabolism as it plays a key role in the central control of insulin release [128]. Drp1-mediated mitochondrial abnormalities have been linked to synaptic injury in the diabetic hippocampus [129]. A clinical trial in patients with T2DM-related cognitive decline observed a decrease in mitochondrial copy number, indicating that decreased mitochondrial biogenesis occurs in DM patients [130]. A decrease in the expression of PGC-1α (peroxisome proliferator-activated receptor-gamma coactivator), TFAM (mitochondrial transcription factor A), and NRFs (nuclear respiratory factors) in diabetic rat brains also corroborates dysfunctional mitochondrial biogenesis [131]. As a result, DM-linked attenuation of mitochondrial biogenesis does not restore decreased mitochondrial mass following the autophagosomal degradation of damaged mitochondria by mitophagy and leads to exacerbation of cellular damage and decline in brain functional ability.

## 7. Conclusions

Fuel and energy homeostasis is maintained by the intricate synchronization of afferent and efferent signals from the periphery and CNS. While the canonical explanation of systemic glucose regulation is founded on the function of exocrine in the pancreas, the homeostatic role of the brain is less clear, particularly in view of insulin-independent regulation of blood glucose. Recent evidence, however, suggests that the pancreas is not a stand-alone regulator but a component of a larger regulatory system integrating other critical homeostatic regulators governed by the brain [26]. The failure of tight cooperation between homeostatic regulators may progress to the pathogenesis of diabetes and contribute to accelerated brain damage, cognitive impairment, and neurodegeneration. As a consequence of the glucoregulatory failure, deregulated energy fluxes affect mitochondria and their functions. During the initial period of DM, the mitochondrial quality program ensures cellular homeostasis through the coordination of the processes of mitochondrial biogenesis, dynamics, mitophagy, and proteasome degradation. However, an inevitable outcome of the chronic nature of diabetes is the failure of adaptive cell programs, resulting in the persistence of oxidative and proteotoxic stress and irreparable alterations in mitochondrial proteome. Proteotoxicity and modified mitochondrial proteomes represent aggravating factors that compromise neuronal cell functions and survival.

Even if intensive research is still warranted to fully understand the impact of diabetes on the brain, the decipherment of the cooperative and controlling mechanisms and molecular mediators implicated in DM-linked brain impairment and neurodegeneration will be of great use for the development of new therapeutic strategies. It is reasonable to assume that effective treatment strategies will be aimed not at one particular target but at a complex interdependent network of targets that will alleviate multiorgan damage in diabetes.
